# Visualization of Ceramide-Associated Proteins in Ceramide-Rich Platforms Using a Cross-Linkable Ceramide Analog and Proximity Ligation Assays With Anti-ceramide Antibody

**DOI:** 10.3389/fcell.2019.00166

**Published:** 2019-08-16

**Authors:** Xue Jiang, Zhihui Zhu, Haiyan Qin, Priyanka Tripathi, Liansheng Zhong, Ahmed Elsherbini, Sanjib Karki, Simone M. Crivelli, Wenbo Zhi, Guanghu Wang, Stefanka D. Spassieva, Erhard Bieberich

**Affiliations:** ^1^Department of Rehabilitation, ShengJing Hospital of China Medical University, Shenyang, China; ^2^Department of Physiology, University of Kentucky, Lexington, KY, United States; ^3^College of Life Sciences, China Medical University, Shenyang, China; ^4^Center for Biotechnology and Genomic Medicine, Medical College of Georgia, Augusta University, Augusta, GA, United States

**Keywords:** ceramide, proximity ligation assay, cross-link, lipid raft, microtubules, mitochondria, VDAC1, acetylated tubulin

## Abstract

Ceramide-rich platforms (CRPs) mediate association of proteins with the sphingolipid ceramide and may regulate protein interaction in membrane contact sites to the cytoskeleton, organelles, and infectious pathogens. However, visualization of ceramide association to proteins is one of the greatest challenges in understanding the cell biology of ceramide. Here we introduce a novel labeling technique for ceramide-associated proteins (CAPs) by combining photoactivated cross-linking of a bioorthogonal and bifunctional ceramide analog, pacFACer with proximity ligation assays (PLAs). pacFACer cross-linked to CAPs is covalently attached to a fluorophore using click chemistry. PLAs use antibodies to: (1) the candidate CAP and the fluorophore (PLA1); and (2) the CAP and ceramide (PLA2). PLA1 shows the subcellular localization of a particular CAP that is cross-linked to pacFACer, while PLA2 tests if the cross-linked CAP forms a complex with endogenous ceramide. Two proteins, tubulin and voltage-dependent anion channel 1 (VDAC1), were cross-linked to pacFACer and showed PLA signals for a complex with ceramide and pacFACer, which were predominantly colocalized with microtubules and mitochondria, respectively. Binding of tubulin and VDAC1 to ceramide was confirmed by coimmunoprecipitation assays using anti ceramide antibody. Cross-linking to pacFACer was confirmed using click chemistry-mediated attachment of biotin and streptavidin pull-down assays. Inhibition of ceramide synthases with fumonisin B1 (FB1) reduced the degree of pacFACer cross-linking and complex formation with ceramide, while it was enhanced by amyloid beta peptide (Aβ). Our results show that endogenous ceramide is critical for mediating cross-linking of CAPs to pacFACer and that a combination of cross-linking with PLAs (cross-link/PLA) is a novel tool to visualize CAPs and to understand the regulation of protein interaction with ceramide in CRPs.

## Introduction

**FIGURE 1:**
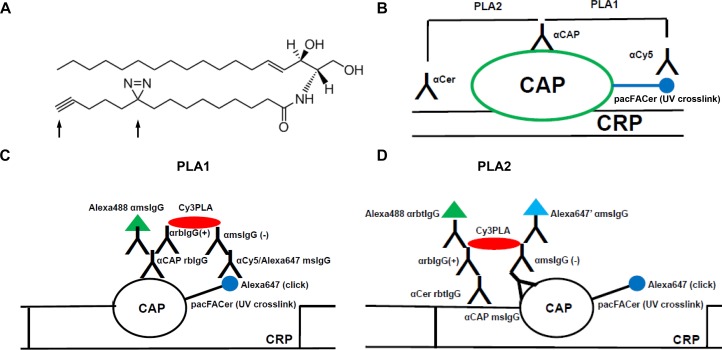
Schematic description of the cross-link/PLA assay. **(A)** Structure of N-(9-(3-pent-4-ynyl-3-H-diazirin-3-yl)-non-anoyl)-D-erythro-sphingosine (pacFACer). Arrows point at the diazirine and alkyne groups used for UV cross-linking and click chemistry-mediated cycloaddition, respectively. **(B)** Overview on PLA1 [using antibodies (anti-cy5/Alexa647 mouse IgG)] to Alexa Fluor 647 conjugated to cross-linked pacFACer and ceramide-associated protein (CAP), and PLA2 (using antibodies to CAP and ceramide). **(C,D)** Details on antibodies used for PLA1 and PLA2. CRP, ceramide-rich platform.

The function of ceramide as precursor and intermediate in sphingolipid metabolism is well investigated for many decades. About 30 years ago, it was shown that ceramide plays an additional role in cell signaling pathways for regulation of cell cycle arrest and apoptosis (Merrill et al., 1986; Mathias et al., 1991; Dobrowsky and Hannun, 1992). Since then, thousands of studies have extended our knowledge on the role of ceramide in many different aspects of cell biology from embryo development to aging and disease. However, to understand the molecular mechanism(s) underlying this plethora of different functions, it is critical to develop methods analyzing the interaction of ceramide with other cellular components, particularly proteins binding to ceramide (Canals et al., 2018). In recent years, novel probes were developed to identify and visualize these ceramide-associated proteins (CAPs). These probes are ceramide analogs that are termed bioorthogonal because labeling of CAPs is achieved in living cells (Walter et al., 2016; Fink and Seibel, 2018). They are also bifunctional in that these ceramide analogs are: (1) cross-linked to CAPs by photoactivation; and (2) can be covalently attached to fluorophores and other functional groups using click chemistry. However, since introduction of the first bioorthogonal, bifunctional ceramide analog, N-(9-(3-pent-4-ynyl-3-H-diazirin-3-yl)-non-anoyl)-D-erythro-sphingosine (pacFACer) (Figure 1A), the number of cross-linked proteins has substantially grown. To understand which of these proteins are actually CAPs mediating the biological function of ceramide, it is critical to develop methods that verify the interaction of the pacFACer cross-linked proteins with endogenous ceramide.

Previously, we showed that pacFACer was UV cross-linked to tubulin in living cells (Kong et al., 2018). Cross-linking was consistent with association of tubulin with ceramide in ceramide-enriched mitochondria-associated membranes (CEMAMs), a MAM subcompartment that was recently discovered by our group (Kong et al., 2018). Our data showed that ceramide-associated tubulin (CAT) regulates opening of the ATP/ADP channel protein VDAC1 in mitochondria. Our results also indicated that additional proteins are cross-linked to pacFACer and colocalized with ceramide, particularly at mitochondria, the cytoskeleton, and the plasma membrane. However, the identity of these proteins is not clear. To test if a particular protein is cross-linked to pacFACer and colocalized with ceramide, we developed a novel labeling technique for visualization of CAPs in cells and tissues using immunocytochemistry.

To achieve labeling of CAPs we first identified candidate proteins using cross-linking to pacFACer followed by click chemistry-mediated covalent attachment of a fluorophore and biotin, ideally as a dual-label click probe such as TAMRA-biotin azide or Cy5-biotin azide. After protein solubilization and copper-catalyzed click reaction, cross-linked protein was isolated using streptavidin pull-down followed by SDS-PAGE and proteomics analysis of fluorescent bands. Based on our previously published data, we focused on two proteins detectable in the proteomics screen: tubulin and its binding partner VDAC1. In addition to CEMAMs, we detected colocalization of ceramide with tubulin at microtubules and the plasma membrane, suggesting that CAT may also partake in the tubulin pool at microtubules and cellular membranes. In particular, membrane tubulin, a minor portion of tubulin associated with cellular membranes, is a candidate for interaction with ceramide in ceramide-rich platforms (CRPs) potentially mediating attachment of ceramide microdomains or rafts to the cytoskeleton (Burgert et al., 2017). Membrane tubulin was shown to regulate channels in cellular membranes, including VDAC1 and the Na/K channel in the plasma membrane (Rostovtseva and Bezrukov, 2008; Rostovtseva et al., 2008; Maldonado and Lemasters, 2012; Kong et al., 2018; Santander et al., 2019). In addition to interacting with CAT, VDAC1 was detected in our initial screen, suggesting that it binds to ceramide itself. However, it is not known if cross-linking to pacFACer is a sufficient indicator for ceramide association of a particular protein in cells. Further, it is also not clear how pacFACer cross-linking and location of CAPs in CRPs is regulated by ceramide.

To test cross-linking of pacFACer with a particular candidate CAP and its distribution to CRPs, cross-linking was combined with proximity ligation assays (PLAs) using antibodies to the CAP and the fluorophore attached to pacFACer (PLA1), and the CAP and ceramide (PLA2). PLAs are based on secondary antibodies covalently attached to oligonucleotide primers that initiate rolling circle PCR and incorporation of fluorescent nucleotides if the distance of primary antibodies is smaller than 40 nm (Weibrecht et al., 2010; Kong et al., 2018). PLA1 shows the subcellular localization of a particular CAP that is cross-linked to pacFACer, while PLA2 tests if the cross-linked CAP forms a complex with endogenous ceramide. We used the novel cross-link/PLA assay to visualize CRP association of (acetylated) tubulin and VDAC1 at membranes, microtubules, and mitochondria, respectively. We confirmed cross-linking of the two CAPs to pacFACer by streptavidin pull-down assays. In addition, coimmunoprecipitation assays using anti-ceramide antibody confirmed that the CAPs were associated with ceramide in cellular membranes. Ceramide depletion using the ceramide synthase inhibitor fumonisin B1 (FB1) indicated that that a microenvironment of endogenous ceramide in CRPs is critical for cross-linking of CAPs to pacFACer. Finally, incubation with amyloid beta (Aβ) peptide enhanced pacFACer cross-linking to VDAC1, suggesting that Aβ induces or facilitates interaction of CAPs with ceramide in CRPs. In summary, our results show that CRPs are critical for mediating cross-linking to pacFACer and that the cross-link/PLA assay is a novel tool to visualize CAPs and protein interaction with ceramide.

## Materials and Methods

### Reagents and Antibodies

Dulbecco Modified Eagle Medium (DMEM) was from Hyclone (Logan, UT, United States), and Fetal Bovine Serum (FBS) was from Atlanta Biologicals (Flowery Branch, GA, United States). Penicillin and streptomycin (cat# 15146) were purchased from GIBCO (Grand Island, NY, United States). HEK293T cells were received from Dr. Lin Mei (Augusta University, Augusta, GA, United States) and Neuro2a (N2a) cells (ATCC CCL-131) were obtained from ATCC (Manassas, VA, United States). Duolink PLA probe anti-rabbit plus (DUO92002), Duolink PLA probe anti-mouse minus (DUO92004), and Duolink detection reagent red (DUO92008) were purchased from Sigma Aldrich (St. Louis, MO, United States). N-(9-(3-pent-4-ynyl-3-H-diazirin-3-yl)-non-anoyl)-D-erythro-shpingosine (pacFACer, cat# 900404) was obtained from Avanti Polar Lipids (Alabaster, AL, United States). Click-iT cell reaction kits (cat# C10269), Alexa Fluor 647 azide (cat# A10277), protease and phosphatase inhibitors (cat# A32961) were from ThermoFisher Scientific (West Columbia, SC, United States); Click chemistry protein reaction buffer kits (cat# 1001) and TAMRA-biotin-azide (cat# 1048-5) were from Click Chemistry Tools (Scottsdale, AZ, United States). SDS-PAGE sample buffer (cat# S3401) was from Sigma-Aldrich (St. Louis, MO, United States). Fumonisin B1 (cat# 62580) was purchased from Cayman Che mical (Ann Arbor, MI, United States). Amyloid β_1–42_ (cat# AS-60479-01) was obtained from Anaspec (Freemont, CA, United States) was dissolved in 1% ammonia and diluted to 1 mg/ml and neutralized in PBS prior to use. Protein A/G magnetic beads (cat# 88803) was from Pierce Biotechnology (Rockford, IL, United States). Antibodies: Anti ceramide rabbit IgG (1:100 for immunocytochemistry; 1:200 for PLAs) was generated in our laboratory (Krishnamurthy et al., 2007). Anti-GAPDH antibody (cat# D16H11, 1:1000) was obtained from Cell Signaling Technology (Danvers, MA, United States). Anti-acetylated tubulin mouse IgG (cat# T6793, 1:3000 immunocytochemistry; 1:5000 for immunoblot and PLAs) was purchased from Sigma-Aldrich (St. Louis, MO, United States). Anti cy5/Alexa Fluor 647 (cat# sc-166896, 1:100 for PLAs) was from Santa Cruz Biotechnology (Dallas, TX, United States). Anti VDAC1 rabbit IgG (cat# ab15895, 1:200 for immunocytochemistry and PLAs; 1:500 for immunoblots) was purchased from abcam (Cambridge, MA, United States). Secondary antibodies conjugated to Alexa or Cy fluorophores were raised in donkey and purchased from the Jackson Laboratory (Bar Harbor, ME, United States).

### Primary Cultures of Astrocytes

All experiments using primary cultures of astrocytes were carried out according to an Animal Use Protocol approved by the Institutional Animal Care and Use Committee at University of Kentucky. Primary glial cells were isolated from brains of P0-P1 C57BL/6 mice following a protocol previously published (Kong et al., 2018). In brief, brains were dissociated in PBS containing 0.1M glucose, passed through a 40 μm filter, and plated in T-25 flasks in DMEM supplemented with 10% fetal bovine serum, and 1% penicillin/streptomycin solution at 37°C in a humidified atmosphere containing 5% CO_2_. After 7 days, adherent cells were passed to 24-well plates containing uncoated glass coverslips and cultured in DMEM as described above.

### Cross-Linking to pacFACer and Click Chemistry-Mediated Conjugation With Fluorophores

N2a cells or primary cultured astrocytes were incubated under protection from light for 30 min in DMEM medium with 5 μM of the bifunctional ceramide analog N-(9-(3-pent-4-ynyl-3-H-diazirin-3-yl)-non-anoyl)-D-erythro-shpingosine (pacFACer, 1:1000 from a stock in ethanol/2% dodecane). Cells were UV irradiated at 365 nm for 15 min at 37°C in a humidified atmosphere containing 5% CO_2_. Cells were further processed under different treatment conditions depending on final labeling requirements. For visualization of cross-linked proteins at their initial subcellular labeling site, cells were fixed with 4% *p*-formaldehyde/0.5% glutaraldehyde for 15 min at room temperature. For visualization of cross-linked proteins after intracellular transport, cells were washed with PBS and then further incubated for 16 h in medium prior to fixation. Following fixation with *p*-formaldehyde/glutaraldehyde, cells were either directly subjected to the click reaction or first permeabilized by treatment with 0.2% Triton X-100 in PBS for 5 min at room temperature, followed by blocking of non-specific binding sites with 3% ovalbumin in PBS for 1 h at 37°C, and then incubation with anti-ceramide antibody for overnight at 4°C. In this case, cells were subjected to a second fixation reaction with *p*-formaldehyde/glutaraldehyde prior to the click reaction. Cells were washed with PBS and then twice with methanol to remove non-cross-linked pacFACer. The click reaction was performed as previously described using the Click-iT Cell Reaction buffer kit (Thermo Fisher Scientific) and Alexa Fluor 647 azide as fluorophore following the protocol provided by the manufacturer.

### pacFACer Cross-Linking and Streptavidin Pull-Down

N2a cells were incubated with pacFACer (5 μM) for 30 min and then UV irradiated at 365 nm for 15 min at 37°C in a humidified atmosphere containing 5% CO_2_. After washing with PBS twice, non-cross-linked pacFACer was removed by washing and fixing cells with ice-cold methanol for 30 min at −20°C. Cells were scraped off in 1 ml of PBS and collected by centrifugation at 15,000 × *g* for 10 min. Cell pellets were solubilized in 400 μl of 1% SDS in PBS containing protease and phosphatase inhibitors and heated for 10 min at 60°C. Insoluble material was removed by centrifugation at 15,000 × *g* for 10 min at room temperature. The click reaction was performed using the protein reaction kit (Click Chemistry Tools) with 50 μM TAMRA-biotin-azide (Click Chemistry Tools) following the protocol provided by the manufacturer. Non-conjugated TAMRA-biotin-azide was removed by precipitating protein with chloroform/methanol as previously described (Kong et al., 2015, 2018). The resulting protein pellets were solubilized with 100 μl of 1% SDS in PBS containing protease and phosphatase inhibitors and heating for 10 min at 60°C. Insoluble material was removed by centrifugation at 15,000 × *g* for 10 min at room temperature. SDS was neutralized by 10-fold dilution of the sample with 1% Triton X-100 in PBS and the sample centrifuged again to remove any residual insoluble material emerging after dilution. Proteins were then incubated with 50 μl of pre-equilibrated NeutrAvidin beads (ThermoFisher Scientific) for overnight at 4°C. Beads were washed twice with 0.1% SDS and 1% Triton X-100 in PBS, and then once with 50 mM Tris–HCl, pH 7.5. Beads were eluted with SDS-sample buffer (Sigma) containing 6 M urea for 10 min at 60°C and protein analyzed by SDS-PAGE and immunoblotting.

### Immunoprecipitation of Ceramide-Enriched Vesicles

Immunprecipitation of ceramide-enriched vesicles was performed using a method established in our laboratory as previously published (He et al., 2012). HEK293T cell pellets were lysed using a Dounce homogenizer in lipid binding buffer [20 mM Tris–HCl, 150 mM NaCl, 1 mM EDTA, pH 7.5, supplemented with protein inhibitor cocktail (Roche)]. Any insoluble debris was removed by centrifugation at 20,800 × *g* for 30 min at 4°C. Lysates (1 ml) were then pre-cleared with Pierce protein A/G magnetic beads (Pierce Biotechnology, CAT#88802, Rockford, United States) and incubated overnight with non-specific rabbit IgG (control) or anti-ceramide rabbit IgG (5 μg/ml) at 4°C under rotational movement. Fifteen microliters of Pierce protein A/G magnetic beads were added to the samples and incubated for 2 h at room temperature under rotational movement. The beads were washed three times using lipid binding buffer. Protein bound to the beads was eluted by SDS-sample buffer and then subjected to SDS-PAGE for immunoblotting using anti-acetylated tubulin mouse IgG or anti-VDAC1 rabbit IgG.

### Proximity Ligation Assays (PLAs) and Immunocytochemistry

N2a cells, HEK293T cells, and primary cultured astrocytes grown on glass cover slips were subjected to the pacFACer cross-linking and click reactions as described and then further analyzed in two distinct PLA assays. PLA1 used primary antibodies against the candidate CAP presumably cross-linked to pacFACer and the fluorophore attached via click addition. In our study, we used anti acetylated tubulin or anti VDAC1 rabbit IgGs and anti Cy5/Alexa Fluor 647 mouse IgG. PLA2 used primary antibodies raised in mouse against the candidate CAP and anti-ceramide rabbit IgG. Accordingly, PLA1 and 2 were performed using anti-rabbit PLUS affinity-purified donkey anti-rabbit IgG (H + L) and anti-mouse MINUS affinity-purified donkey anti-mouse IgG (H + L). This reaction and the consecutive ligation and amplification steps were performed following the protocol provided by the manufacturer [Duolink PLA kit red (Texas Red) from Sigma-Aldrich] as previously described (Kong et al., 2018). After completion of the PLA1 and 2 reactions, samples were post-incubated with secondary antibodies conjugated to fluorophores in unused fluorescence channels to visualize the intracellular distribution of antigens tested in the PLA assay. Epifluorescence microscopy was performed with a Nikon Ti2 Eclipse microscope equipped with NIS Elements software using Z-scanning with 60× or 100× oil immersion objectives at the step size (0.2–0.3 μm) recommended by the software. Images were processed using the NIS Elements 3D deconvolution program at settings recommended by the software (automated) and PLA signals counted using NIS Elements and NIH Image J software. Images shown in figures are 3D rendered Z-scans. For counting, Z-scan images were collapsed onto one plane and thresholds for pixel size and fluorescence intensity defined based on comparison with non-deconvolved images to represent authentic signals. All of the data were collected by laboratory personnel blinded to the origin of the samples from at least three independent cell cultures using at least five randomly selected areas/culture. Images obtained with secondary antibody only were used as negative controls.

### Statistical Analysis

Statistical significance was calculated using Student’s *t*-test with or one-way ANOVA and Tukey’s *post hoc* with GraphPad Prism. *P* < 0.05 was considered significant.

### Miscellaneous

Protein concentrations of input used for pull-down experiments were determined using the RD/DC protein assay (Bio Rad), following a protocol provided by the manufacturer. For immunoblot analysis, equal amounts of protein were loaded onto a 10% denaturing and reducing SDS gel and SDS-PAGE was performed using the Laemmli method. For the immunoblotting reaction, membranes were first blocked with 3% BSA in PBST (PBS containing 0.1% Tween-20) and incubated with primary antibodies in blocking buffer overnight at 4°C. Membranes were then washed three times with PBST and incubated with the appropriate horseradish peroxidase-conjugated secondary antibodies for 1 h at room temperature. After washing, bands were detected using the Pico or Femto ECL assay from Pierce/Thermo Fisher Scientific and Azure imager.

## Results

### Cross-Linking/PLA Using pacFACer – Principle of Method

To test association of a cross-linked protein with CRPs, cells were incubated with pacFACer and exposed to UV radiation. Cells were either fixed with para-formaldehyde/glutaraldehyde to identify the initial cellular site of pacFACer cross-linking, or alternatively, they were further incubated to monitor intracellular transport and distribution of the cross-linked protein over time. After fixation, two sets of cells underwent copper-mediated click chemistry reactions to attach the Alexa Fluor 647 fluorophore, which were followed by two distinct PLAs (Figure 1B). For the first PLA reaction (PLA1), fixed cells were directly subjected to the click reaction and then incubated with antibodies to perform the PLA1 reaction (Figure 1C). For the second PLA reaction (PLA2), cells were first incubated with antibodies – one of which was anti-ceramide antibody – and then fixed again prior to the click reaction (Figure 1D). The second fixation step was critical to keep anti-ceramide antibody in place since click chemistry requires wash steps with organic solvent that would remove ceramide as antigen from cellular membranes.

The PLAs were used for *in situ* visualization of antigen complexes using fluorescence microscopy. PLA1 used a combination of antibodies to a potential CAP and the fluorophore attached by the click reaction. This PLA reaction tested if pacFACer was cross-linked to the CAP of interest. PLA1 was complemented by pull-down assays with streptavidin beads after using biotin azide for cross-linking. PLA2 used a combination of antibodies to the specific CAP and ceramide. This PLA reaction tested if the CAP cross-linked to pacFACer formed a complex with endogenous ceramide. PLA2 was complemented by coimmunoprecipitation assays using anti-ceramide antibody.

The cross-link/PLA assay was supplemented by additional colabeling experiments and PLA reactions with or without click addition of the fluorophore. For example, the PLA1 reaction was combined with antibodies against organelle markers to identify the subcellular localization of the cross-linked CAP. When omitting click addition or even crosslinking, PLA2 was followed by incubation with fluorescent secondary antibodies to the primary antibodies used for PLA. This reaction visualized the intracellular distribution of the complex between the candidate CAP and endogenous ceramide within the entire pool of ceramide and the CAP. It is important that all secondary antibodies used for PLA or immunofluorescence were raised in a species distinct from that of any of the primary antibodies. Alternatively, kits are commercially available for direct labeling of the primary antibodies with oligonucleotides for PLA or fluorophores for immunofluorescence microscopy.

### pacFACer Is Cross-Linked to Tubulin and VDAC1 at Microtubules and Mitochondria, Respectively

**FIGURE 2:**
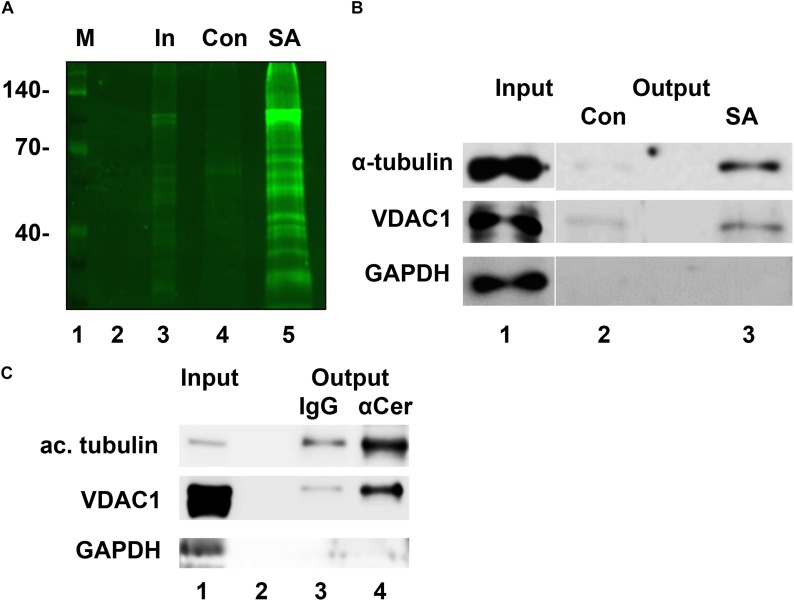
Tubulin and VDAC1 are cross-linked to pacFACer and associated with ceramide vesicles. **(A)** N2a cells were incubated with pacFACer, UV cross-linked, and fixed with ice-cold methanol. Cells were scraped off, pelleted, and protein solubilized with 1% SDS in PBS. The copper-mediated click addition was performed using TAMRA-biotin-azide and non-incorporated reagent removed by chloroform/methanol extraction. Precipitated protein was re-solubilized and pacFACer cross-linked protein isolated by pull-down with streptavidin beads. Bound protein was eluted by heating in SDS sample buffer and separated by SDS gelectrophoresis. Lane 1, marker; lane 2, empty; lane 3, input used for streptavidin pull-down; lane 4, pull-down with streptavidin beads saturated with biotin (control); lane 5, pull-down with streptavidin beads (SA). **(B)** Assay performed as described in **(A)**, but gel immunoblotted and probed with antibodies to tubulin, VDAC1, and GAPDH. **(C)** Coimmuniprecipitation of acetylated tubulin and VDAC1 using antibody to ceramide to precipitate ceramide-enriched vesicles from lysates of HEK293T cells (lane 4). Non-specific rabbit IgG was used as the control (lane 3). Lane 1, input; lane 2, empty. GAPDH was not coimmunoprecipitated.

pacFACer was UV cross-linked to proteins in intact cells and then covalently attached to a fluorescent dye that also carries a biotin group (TAMRA-biotin-azide). Streptavidin pull-down/fluorescent blot analysis showed that a variety of proteins were cross-linked to pacFACer (Figure 2A). Our previous studies – including proteomics analyses of ceramide-binding proteins – indicated that tubulin and VDAC1 are among these proteins, which was confirmed by immunoblot analyses (Figure 2B and see Supplementary Figure 1 for original images from immunoblots) (Kong et al., 2018). The housekeeping protein GAPDH was not among the pulled-down proteins demonstrating the specificity of the pacFACer cross-linking reaction. The significance of pacFACer cross-linking for identifying binding of tubulin and VDAC1 to endogenous ceramide was tested by coimmunoprecipitation of the two proteins using anti-ceramide IgG. Previously, we introduced this method to isolate ceramide-enriched vesicles from cell lysates and identify proteins associated with these vesicles (He et al., 2012; Kong et al., 2018; Zhu et al., 2019). Figure 2C shows that acetylated tubulin and VDAC1 were coimmuniprecipitated with anti-ceramide IgG confirming that these two proteins are associated with endogenous ceramide (see Supplementary Figure 1 for original images from immunoblots).

**FIGURE 3:**
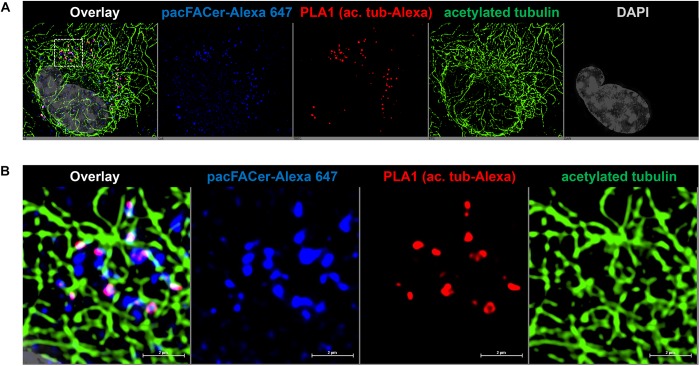
PLA1 reaction using antibodies against acetylated tubulin and Alexa Fluor 647. **(A)** Primary cultured astrocytes were subjected to the PLA1 reaction (fluorescence signals for complexes of acetylated tubulin with Alexa Fluor 647-pacFACer are pseudo-colored in red) and then post-incubated with Alexa Fluor 488-conjugated secondary antibody to visualize microtubules (pseudo-colored in green). Cross-linked pacFACer (conjugated to Alexa Fluor 647) is pseudo-colored in blue. **(B)** Detail from **(A)** shows microtubules with acetylated tubulin cross-linked to pacFACer.

**FIGURE 4:**
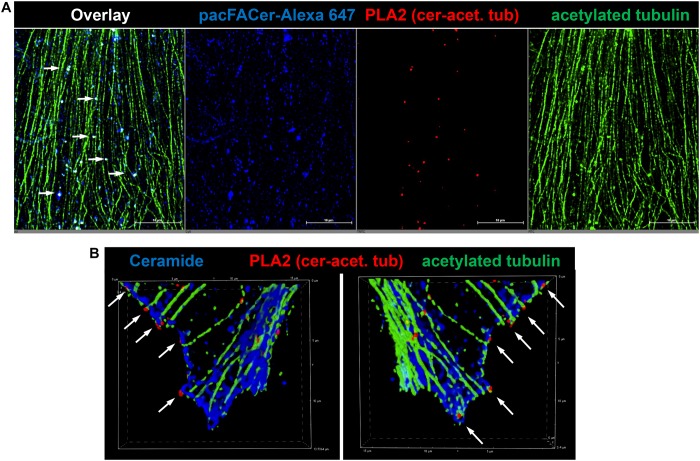
PLA2 reaction using antibodies against acetylated tubulin and ceramide. **(A)** Primary cultured astrocytes were subjected to the PLA2 reaction (fluorescence signals for complexes of acetylated tubulin with ceramide are pseudo-colored in red) and then post-incubated with Alexa Fluor 488-conjugated secondary antibody to visualize microtubules (pseudo-colored in green). Cross-linked pacFACer (conjugated to Alexa Fluor 647) is pseudo-colored in blue. **(B)** PLA2 reaction performed as in **(A)**, but pacFACer not conjugated to Alexa Fluor 647. The PLA2 reaction was post-incubated with Alexa Fluor 488-conjugated anti mouse IgG to visualize acetylated tubulin (pseudo-colored in green) and Alexa Fluor 647-conjugated anti-rabbit IgG to visualize ceramide (pseudo-colored in blue).

**FIGURE 5:**
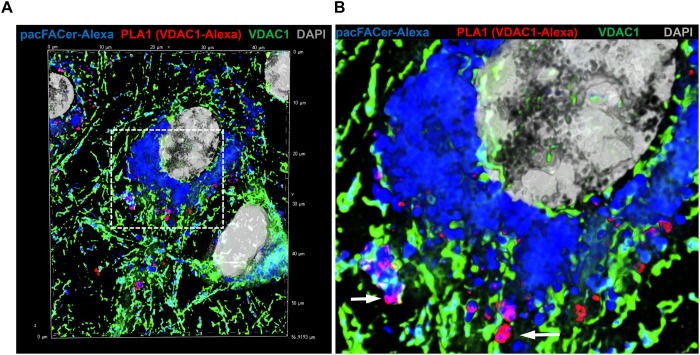
PLA1 reaction using antibodies against VDAC1 and Alexa Fluor 647. **(A)** Primary cultured astrocytes were subjected to the PLA1 reaction (fluorescence signals for complexes of VDAC1 with Alexa Fluor 647-pacFACer are pseudo-colored in red) and then post-incubated with Alexa Fluor 488-conjugated anti-rabbit IgG to visualize mitochondria (pseudo-colored in green). Cross-linked pacFACer (conjugated to Alexa Fluor 647) is pseudo-colored in blue. **(B)** Detail from **(A)** shows mitochondria with VDAC1 cross-linked to pacFACer.

Next, we tested if acetylated tubulin and VDAC1 were cross-linked to pacFACer at microtubules and mitochondria, respectively. Acetylated tubulin was chosen because of our previously published studies showing colocalization of ceramide with acetylated tubulin (He et al., 2014; Kong et al., 2015, 2018). Figures 3A,B shows that the fluorescence signal for cross-linked pacFACer (visualized by attaching Alexa Fluor 647) was colocalized with the PLA1 signal (Texas Red fluorescence) for a complex of pacFACer and acetylated tubulin at microtubules (visualized by Alexa Fluor 488 for acetylated tubulin). The PLA1 reaction was also performed with the cell types used for the pull-down assays (N2a and HEK293T cells, see Supplementary Figure 2), confirming results obtained with primary cultured astrocytes (Figure 3). Figures 4A,B shows the results of the PLA2 reaction using antibodies to acetylated tubulin and ceramide (see also Supplementary Figure 3). The results of the PLA2 reaction indicated that ceramide formed a complex with acetylated tubulin cross-linked to pacFACer at microtubules (arrows in Figure 4A). In addition, complexes for ceramide and acetylated tubulin were detected at the plasma membrane, particularly at microtubule attachment sites (arrows in Figure 4B). Figures 5A,B shows that cross-linked pacFACer was also colocalized with the PLA1 reaction for pacFACer and VDAC1 at mitochondria (see Supplementary Figure 2 for PLA1 with N2a and HEK293T cells). The PLA2 reaction could not be reliably performed because of some degree of non-specific reactions with anti VDAC1 mouse IgG.

### pacFACer Cross-Linking of Tubulin and VDAC1 Is Decreased by FB1 and Increased by Aβ

**FIGURE 6:**
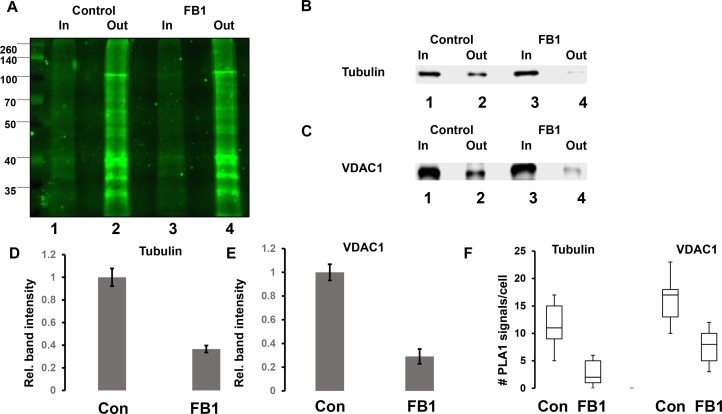
Ceramide depletion reduces cross-linking of pacFACer to tubulin and VDAC1. **(A)** N2a cells with or without pre-incubation for 48 h with 5 μM FB1 were treated with pacFACer and cross-linked protein isolated and separated by SDS-PAGE as described in the legend for Figure 2. Lane 1, input for pull-down of pacFACer cross-linked protein from non-treated control cells; lane 2, output from pull-down; lane 3, input for pull-down of pacFACer cross-linked protein from FB1-treated cells; lane 4, output from pull-down. **(B,C)** Assay performed as described in **(A)**, but gel immunoblotted and probed with antibodies to tubulin **(B)** and VDAC1 **(C)**. **(D)** Quantitation of **(B)**. *n* = 7, *P* < 0.05. **(E)** Quantitation of **(C)**. *n* = 5, *P* < 0.005. **(F)** Quantitation of PLA1 signals for pacFACer cross-linked tubulin and VDAC1 in primary cultured astrocytes with or without ceramide depletion by incubation with FB1. *n* = 5, *P* < 0.05.

The colocalization of pacFACer cross-linked tubulin and VDAC1 with ceramide suggested that a ceramide-enriched membrane environment affects the cross-linking reaction. To test the effect of ceramide on pacFACer cross-linking we pre-incubated N2a cells and primary cultured astrocytes with fumonisin B1 (FB1), a fungus toxin specifically inhibiting ceramide synthase and reliably decreasing the ceramide concentration in a variety of cell types (Wang et al., 2009; Stiban et al., 2010; Lee et al., 2011; Kong et al., 2018). Figures 6A–E shows that ceramide depletion with FB1 reduced the amount of pacFACer cross-linked tubulin and VDAC1, suggesting that endogenous ceramide mediated the cross-linking reaction to pacFACer. This conclusion was confirmed by PLA1 assays after pre-incubation with FB1 [Figure 6F and see Supplementary Figure 1 (original images of immunoblots), 4 (fluorescence images) for details on quantitation shown in Figure 6], showing that the number of PLA signals for pacFACer cross-linked tubulin and VDAC1 was reduced by 50–70%.

**FIGURE 7:**
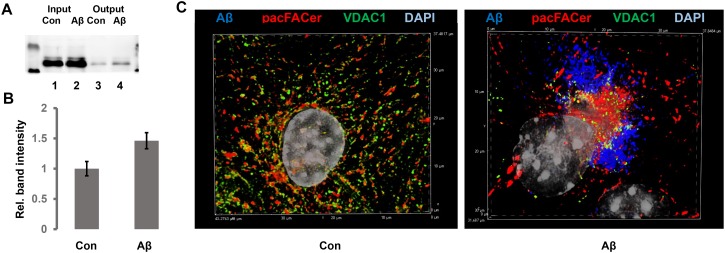
Aβ enhances cross-linking of pacFACer to VDAC1. **(A)** N2a cells with or without pre-incubation for 24 h with 1 μM Aβ_42_ were treated with pacFACer and cross-linked protein isolated and separated by SDS-PAGE, followed by immunoblotting using antibody to VDAC1. Lane 1, input for pull-down of pacFACer cross-linked protein from non-treated control cells; lane 2, input for pull-down of pacFACer cross-linked protein from Aβ_42_-treated cells; lane 3, output from pull-down control; lane 4, output from pull-down Aβ. **(B)** Quantitation of **(A)**. *n* = 6, *P* < 0.005. **(C)** Immunocytochemistry of pacFACer cross-linked astrocytes (pseudo-colored in red) with or without Aβ treatment. VDAC1 was labeled with anti-VDAC1 rabbit IgG and Alexa Fluor 488 anti-rabbit IgG (pseudo-colored in green) and Aβ_42_ was labeled with anti-Aβ_42_ mouse IgG (clone 4G8, pseudo-colored in blue).

In addition to direct interference with ceramide generation, we incubated N2a cells and primary astrocytes with Aβ to test the effect on pacFACer cross-linking of tubulin and VDAC1. We chose Aβ because it is known to increase the ceramide level in astrocytes and to interact with tubulin and VDAC1 (Manczak and Reddy, 2012; Wang et al., 2012; Fernandez-Echevarria et al., 2014; Saha et al., 2015; Smilansky et al., 2015). Our previous studies also showed that it is an infectious protein the spreading of which is mediated by association with ceramide-enriched exosomes (Wang et al., 2012; Dinkins et al., 2016a, b). Figures 7A,B shows that overnight incubation with 1 μM oligomeric Aβ increased the amount of pacFACer cross-linked VDAC1, suggesting that Aβ enhanced the effect of ceramide on facilitating cross-linking to pacFACer (see Supplementary Figure 1 for original images of immunoblots shown in Figure 7). Consistent with the pull-down assay, immunocytochemistry showed that Aβ induced colocalization between VDAC1 and cross-linked pacFACer as well as ceramide (Figure 7C). In summary, our results indicated that the extent of pacFACer cross-linking to VDAC1 was correlated with the extent of ceramide interaction.

## Discussion

Ceramide is a sphingolipid the interaction of which with proteins is implicated in a plethora of physiological and pathophysiological processes. In cellular membranes, it is enriched in microdomains or rafts, currently referred to as CRPs (Burgert et al., 2017; Bieberich, 2018). CRPs are suggested to form contact sites with the cytoskeleton, intracellular membranes and organelles, and infectious proteins, cells, and viruses. One of the greatest challenges in determining the function of CRPs is to visualize the interaction of ceramide with proteins in these contact sites. Since pacFACer is taken up by living cells, cross-linking can be used to identify and visualize proteins potentially associated with ceramide. We and others used this method to visualize pacFACer cross-linked tubulin and other CAPs (Kong et al., 2015, 2018; Dinkins et al., 2016a; Bockelmann et al., 2018). However, there are two major limitations to this method. When using photoactivatable sphingolipid analogs for labeling in fluorescence microscopy, the first limitation arises from the spatial resolution of the fluorescence signal, which is 200 nm at best. The spatial resolution can be improved up to 10-fold using superresolution microscopy (Burgert et al., 2017). However, there is still the possibility that the CAP is co-localized with the cross-linked protein, but not identical to it. Therefore, visualization of cross-linked CAPs using colabeling in immunofluorescence microscopy should be complemented by another method such as cross-linking to biotin and pull-down with immobilized streptavidin, followed by immunoblotting or proteomics analysis of the CAP. Unfortunately, there is not always sufficient amount of protein to perform this type of analysis and pull-down from a cell homogenate does not mean that a colabeled protein is also cross-linked in a complex with ceramide visualized by immunocytochemistry. Recent studies addressed a similar problem in the context of visualizing palmitoylated proteins using incorporation of a clickable palmitic acid analog (Gao and Hannoush, 2014, 2016). After attaching the fluorophore to protein-linked palmitic azide using click chemistry, PLA was performed using antibodies against the fluorophore and the presumably palmitoylated protein. We modified this approach using Alexa Fluor 647 azide clicked to cross-linked pacFACer and anti-Cy5/Alexa647 mouse IgG for the PLA reaction with antibody against the presumed CAP. In our assays to detect pacFACer cross-linked tubulin and VDAC1, this reaction is referred to as PLA1.

The second limitation of cross-linking sphingolipid analogs is the lack of information on colocalization of the cross-linked protein with the actual sphingolipid in cells. It is not clear if a protein cross-linked to pacFACer colocalizes or associates with endogenous ceramide. This limitation mainly arises from the non-fixability of lipids with *p*-formaldehyde or incompatibility of membrane lipids with fixation using organic solvents such as methanol or acetone. We developed a protocol bypassing this problem by fixing the anti-ceramide antibody instead of the lipid. Using clickable (not-crosslinked) ceramide analogs we demonstrated that this method reliably labels the subcellular distribution of endogenous ceramide (Walter et al., 2016). The colocalization of the pacFACer cross-linked protein with ceramide is tested in the PLA2 reaction. We show that any PLA reaction can be complemented by post-incubation with a second set of secondary antibodies conjugated to fluorophores. This reaction visualizes the distribution of the CAP and ceramide and at the same time, localization of their complexes. Combining PLA with the click addition of a fluorophore to pacFACer visualizes distribution of the cross-linked protein within CRPs. In our study, PLA2 showed complexes of cross-linked tubulin and VDAC1 with ceramide that are localized in CRPs, suggesting that ceramide affects the cross-linking reaction or distribution of the cross-linked CAP.

Ceramide can affect the cross-linking reaction of a CAP to pacFACer in two ways: it can either compete with pacFACer for the binding site to ceramide or it can facilitate cross-linking by embedding pacFACer in CRPs. These opposite effects were tested by depleting astrocytes and N2a cells of ceramide with fumonisin B1 (FB1), a fungus toxin specifically inhibiting ceramide synthases. The pull-down assays showed that FB1 reduced the amount of cross-linked tubulin and VDAC1 suggesting that ceramide facilitates cross-linking of CAPs to pacFACer, probably by embedding pacFACer into the ceramide microenvironment of CRPs. This conclusion is consistent with the observation that other ceramide analogs are embedded into CRPs when exogenously administered to cells (Walter et al., 2016). While these results cannot demonstrate the role of ceramide binding for the function of a particular protein, they suggest that cross-linking to pacFACer and monitoring complex formation of the cross-linked protein with ceramide (cross-link/PLA assay) can be used to probe the distribution of CAPs to CRPs.

We used the cross-link/PLA assay to test the effect of Aβ on pacFACer cross-linking to VDAC1. Our results showed that Aβ increased the amount of cross-linked VDAC1, suggesting that it is embedded into CRPs promoting the pacFACer cross-linking reaction. Most recently, we reported that ceramide-associated tubulin (CAT) forms a complex with VDAC1 that blocks ADP/ATP transport through the outer mitochondrial membrane (Kong et al., 2018). Results in this study suggest that ceramide association of VDAC1 takes part in this process. In addition to pacFACer cross-linking, ceramide association of VDAC1 is consistent with the observation that it is coimmunoprecipitated with anti ceramide antibody. We also showed that Aβ incubation of astrocytes led to clustering of mitochondria, which was confirmed in the studies presented here. It is currently not known if Aβ first binds to VDAC1 and enhances cross-linking to pacFACer or if Aβ initially associates with CRPs and then enhances pacFACer cross-linking of VDAC1 (Gulbins et al., 2004; Bollinger et al., 2005; Grassme et al., 2008). It is also not known if other VDAC isoforms associate with CRPs. Most recently, VDAC2 was found to bind to ceramide and cross-link to pacFACer (Dadsena et al., 2019), suggesting that several CAPs are cross-linked to pacFACer after embedding into CRPs. In summary, our data show that the novel labeling technique (cross-link/PLA) is a valuable tool to visualize CAPs in CRPs, which will also help determine the function of ceramide in contact sites to infectious proteins and other pathogens.

## Data Availability

All datasets generated for this study are included in the manuscript and/or the Supplementary Files.

## Ethics Statement

All experiments using primary cultures of astrocytes were carried out according to an Animal Use Protocol approved by the Institutional Animal Care and Use Committee at the University of Kentucky.

## Author Contributions

XJ performed the key experiments (cross-linking and click chemistry) and wrote the critical parts of the manuscript. ZZ also performed the click chemistry experiments. PT performed the pull-down with anti-ceramide antibody. LZ and HQ performed the FB1 incubation. AE, SK, and SC performed the *in vitro* experiments with amyloid. WZ performed the proteomics analysis. GW and SS wrote parts of the manuscript. EB performed parts of the microscopy experiments and PLAs, and wrote parts of the manuscript.

## Conflict of Interest Statement

The authors declare that the research was conducted in the absence of any commercial or financial relationships that could be construed as a potential conflict of interest.
