# Supplementary material for: Visualization of Ceramide-Associated Proteins in Ceramide-Rich Platforms Using a Cross-Linkable Ceramide Analog and Proximity Ligation Assays With Anti-ceramide Antibody
Source: Front Cell Dev Biol. 2019 Aug 16;7:166. doi: 10.3389/fcell.2019.00166 (PMC6706757; doi:10.3389/fcell.2019.00166)
Supplement: Supplementary file 1 [file Presentation_1.pptx]

## Slide 1
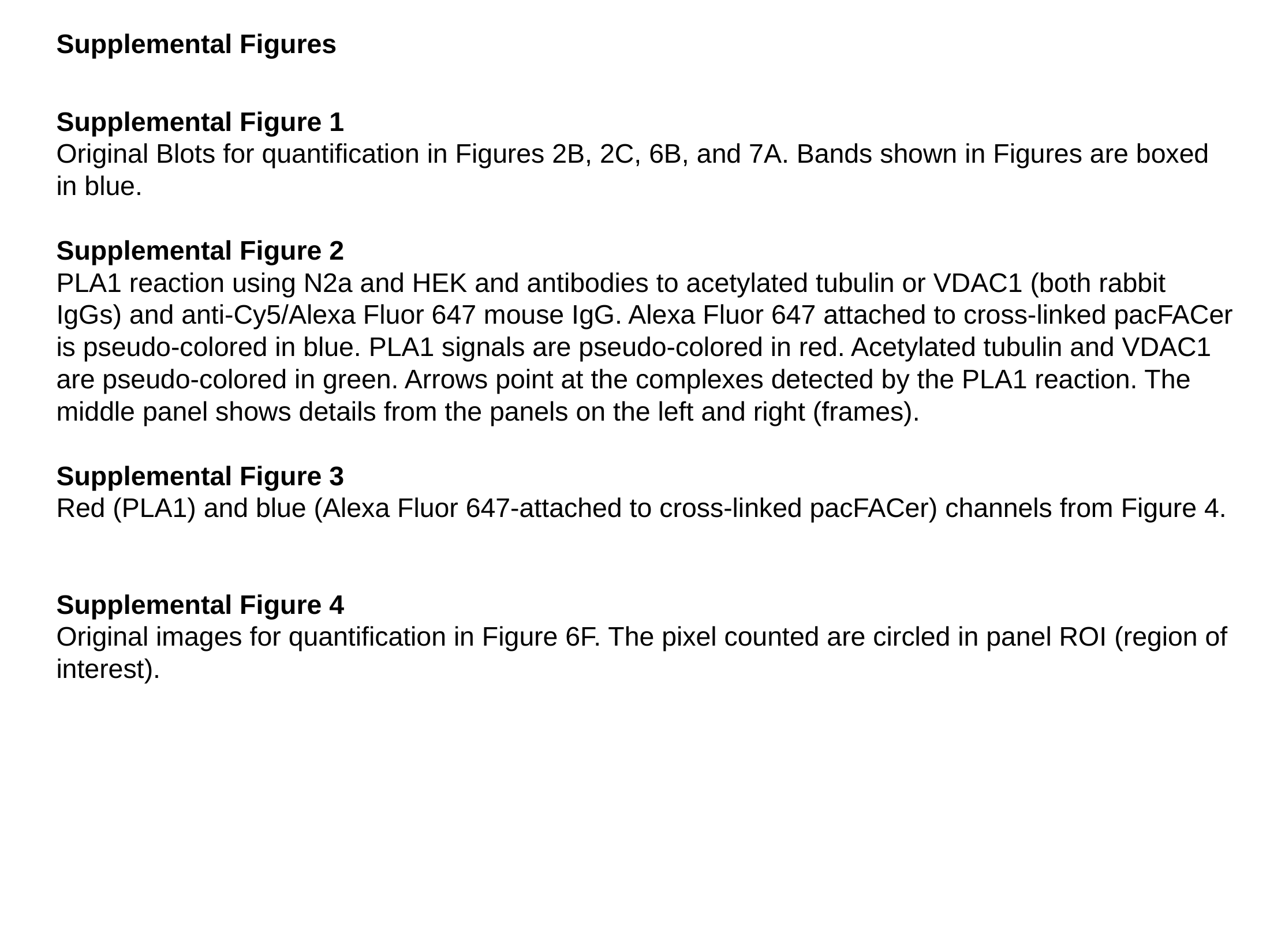

Supplemental Figures
Supplemental Figure 1
Original Blots for quantification in Figures 2B, 2C, 6B, and 7A. Bands shown in Figures are boxed in blue.
Supplemental Figure 2
PLA1 reaction using N2a and HEK and antibodies to acetylated tubulin or VDAC1 (both rabbit IgGs) and anti-Cy5/Alexa Fluor 647 mouse IgG. Alexa Fluor 647 attached to cross-linked pacFACer is pseudo-colored in blue. PLA1 signals are pseudo-colored in red. Acetylated tubulin and VDAC1 are pseudo-colored in green. Arrows point at the complexes detected by the PLA1 reaction. The middle panel shows details from the panels on the left and right (frames).
Supplemental Figure 3
Red (PLA1) and blue (Alexa Fluor 647-attached to cross-linked pacFACer) channels from Figure 4.
Supplemental Figure 4
Original images for quantification in Figure 6F. The pixel counted are circled in panel ROI (region of interest).

## Slide 2
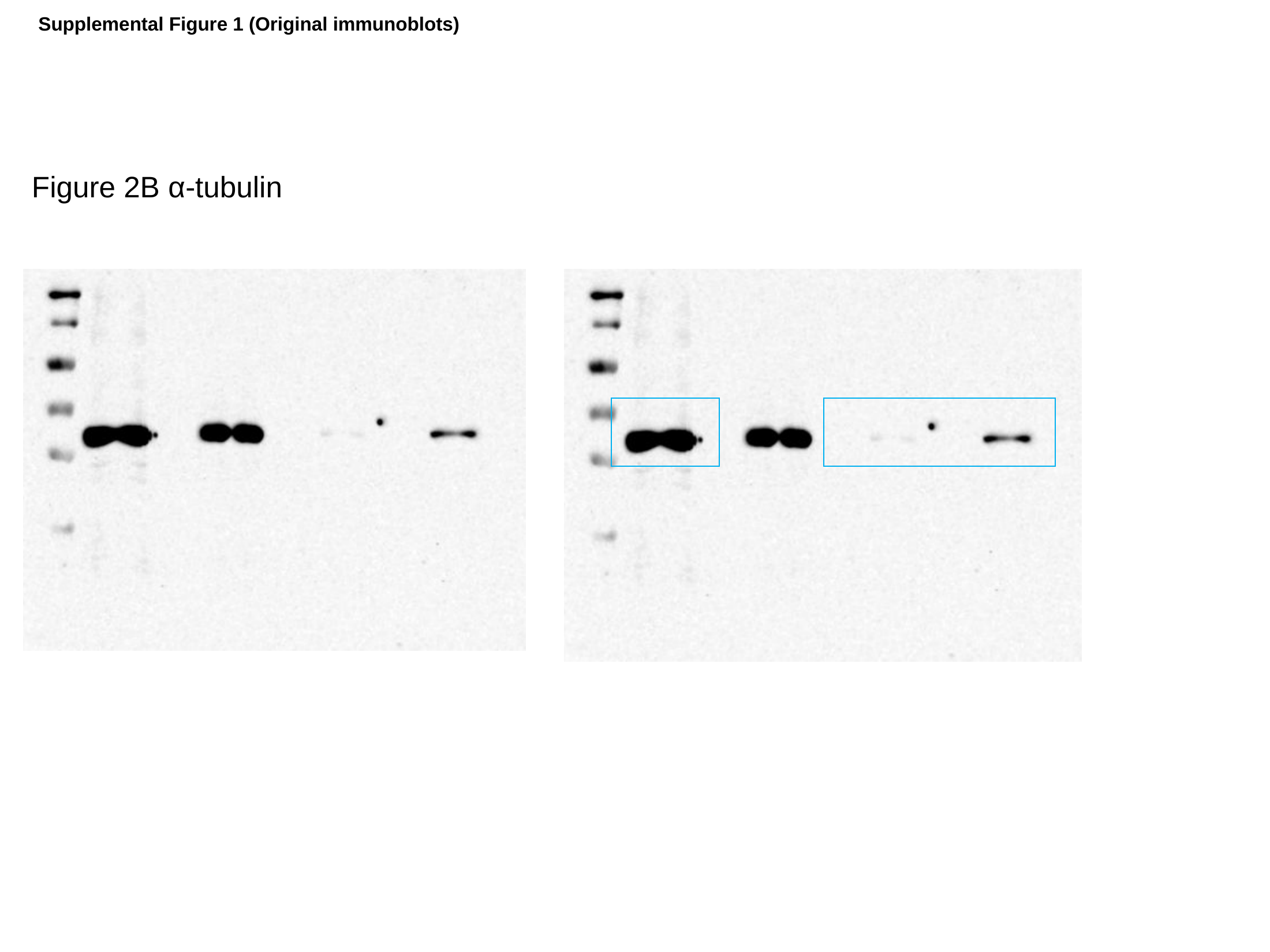

Supplemental Figure 1 (Original immunoblots)
Figure 2B α-tubulin

## Slide 3
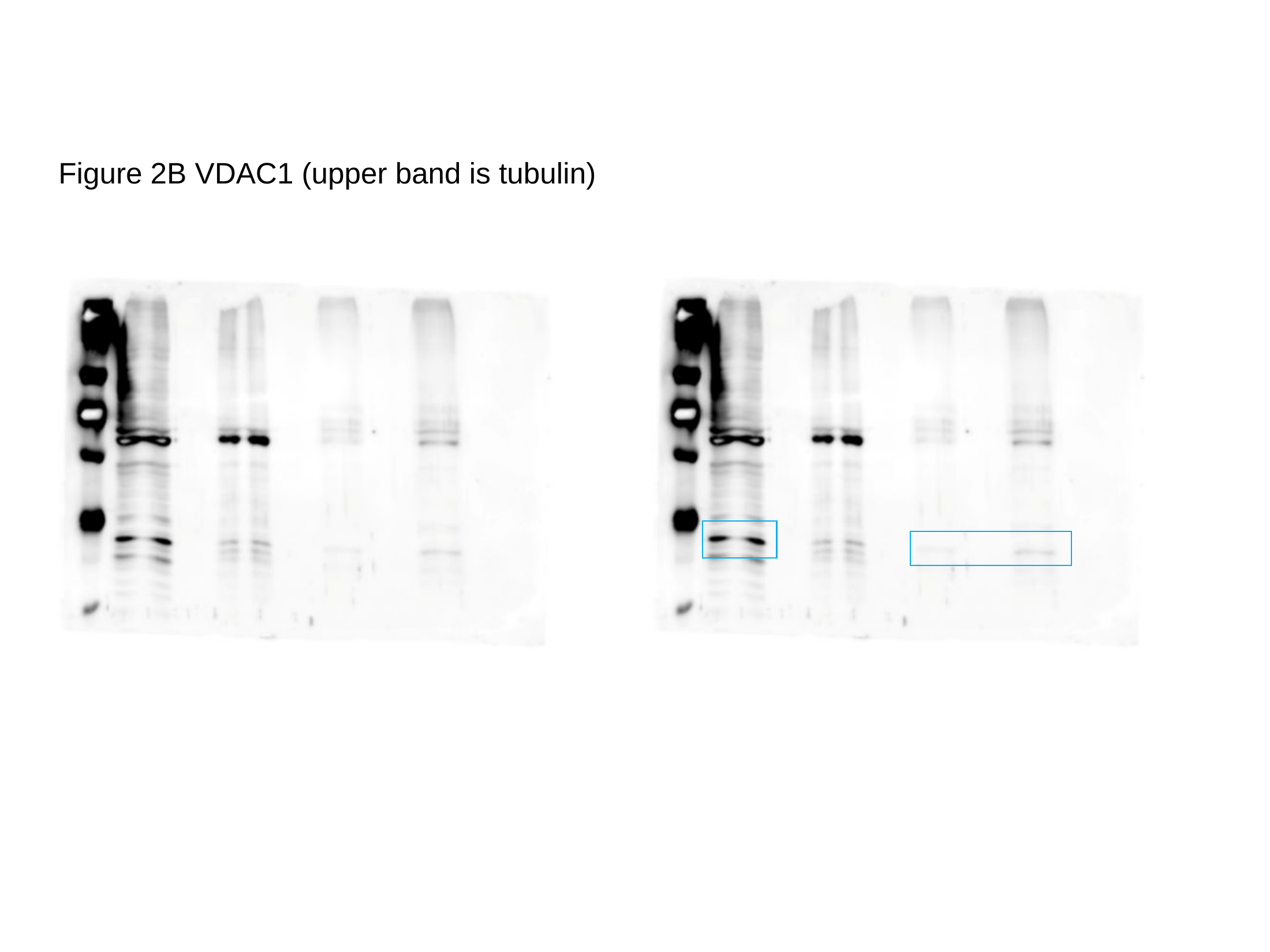

Figure 2B VDAC1 (upper band is tubulin)

## Slide 4
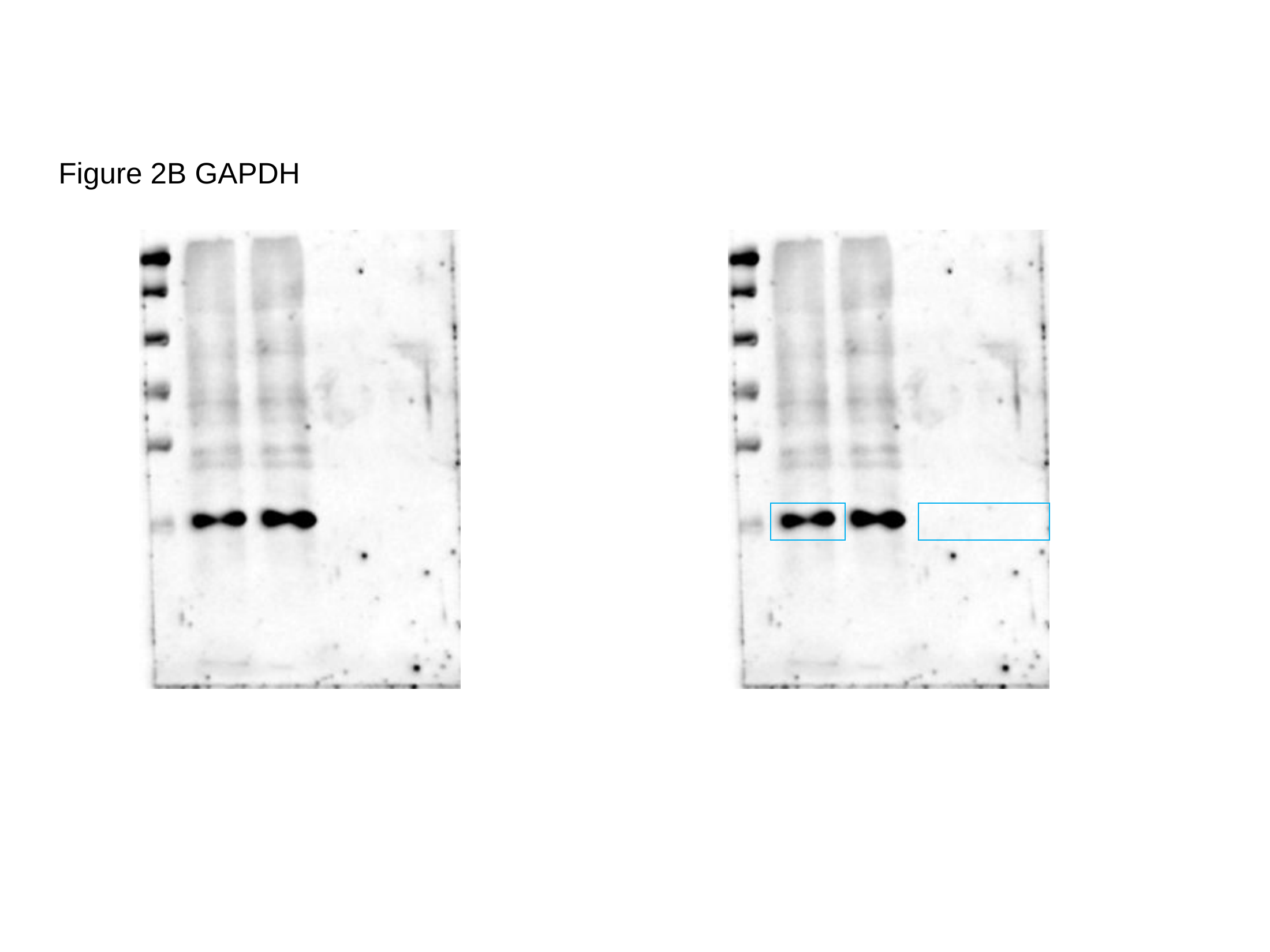

Figure 2B GAPDH

## Slide 5
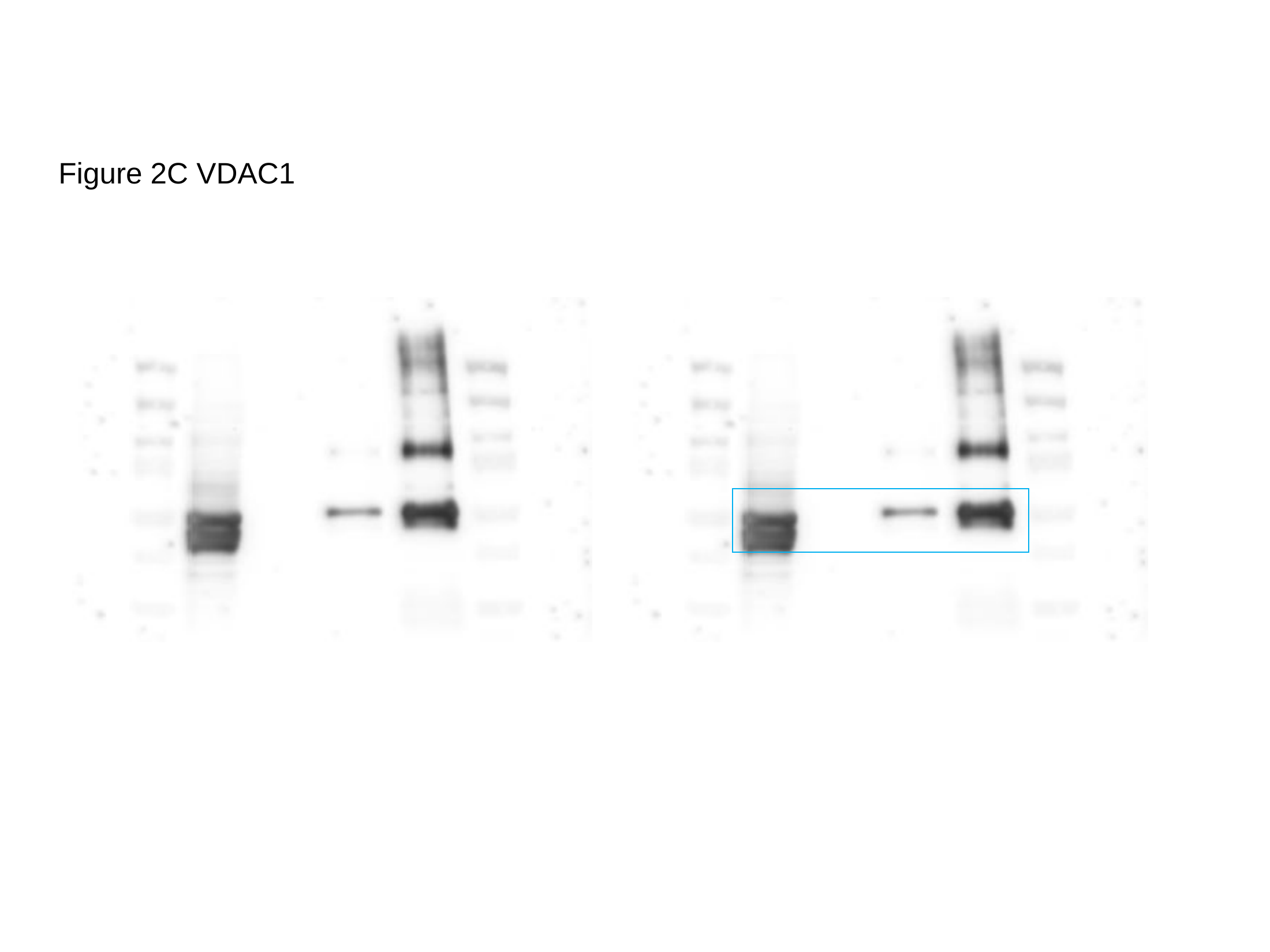

Figure 2C VDAC1

## Slide 6
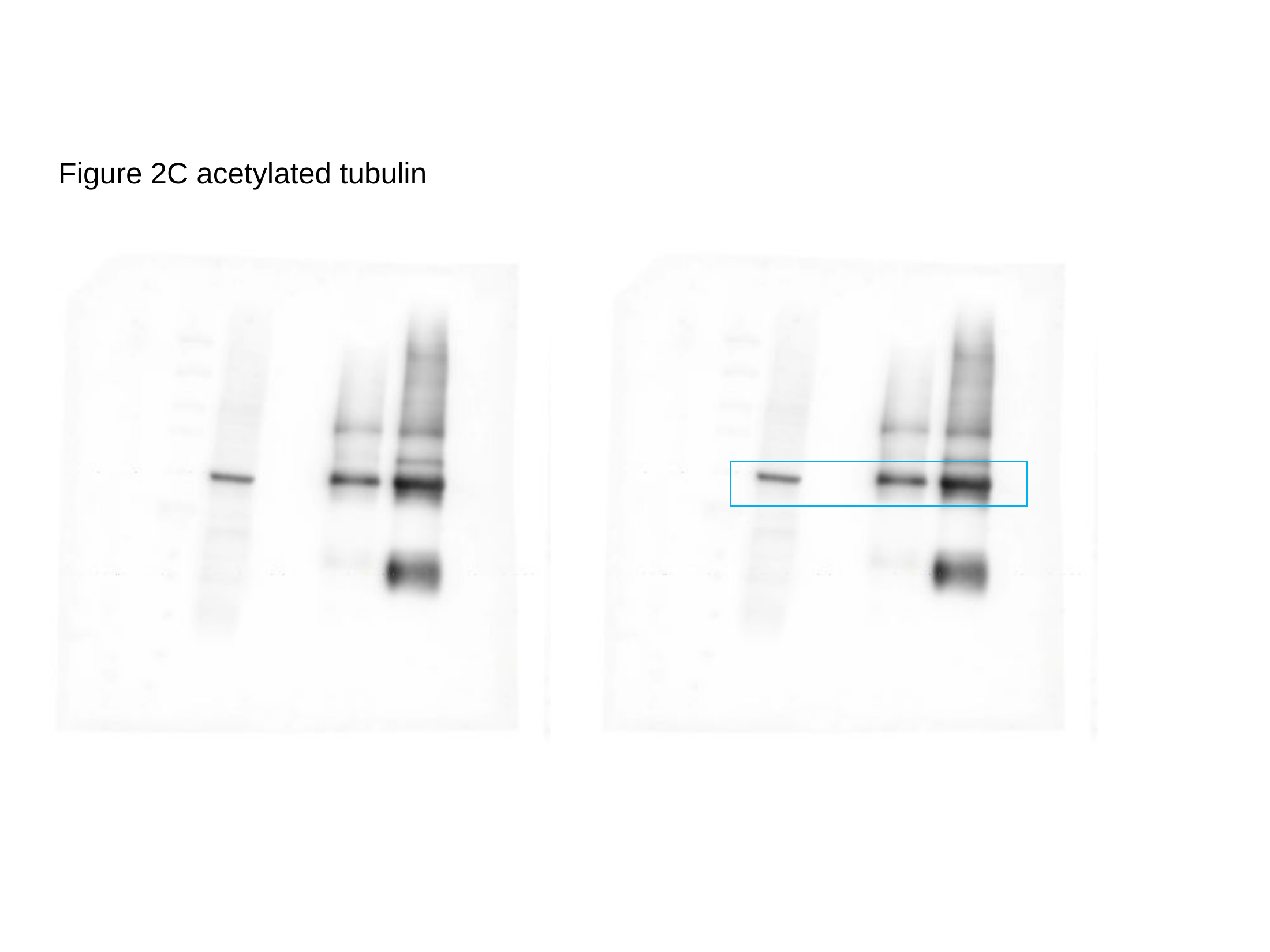

Figure 2C acetylated tubulin

## Slide 7
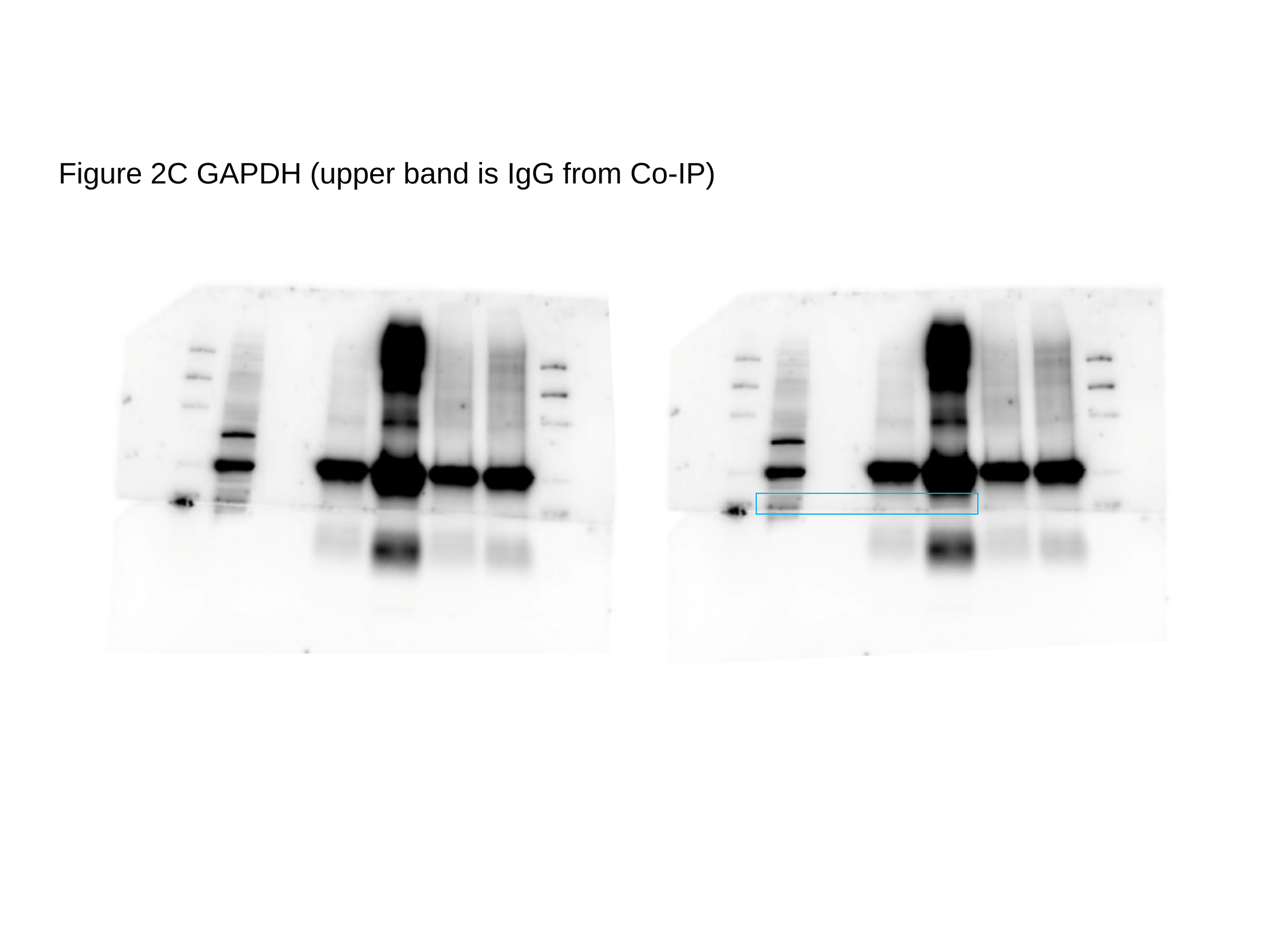

Figure 2C GAPDH (upper band is IgG from Co-IP)

## Slide 8
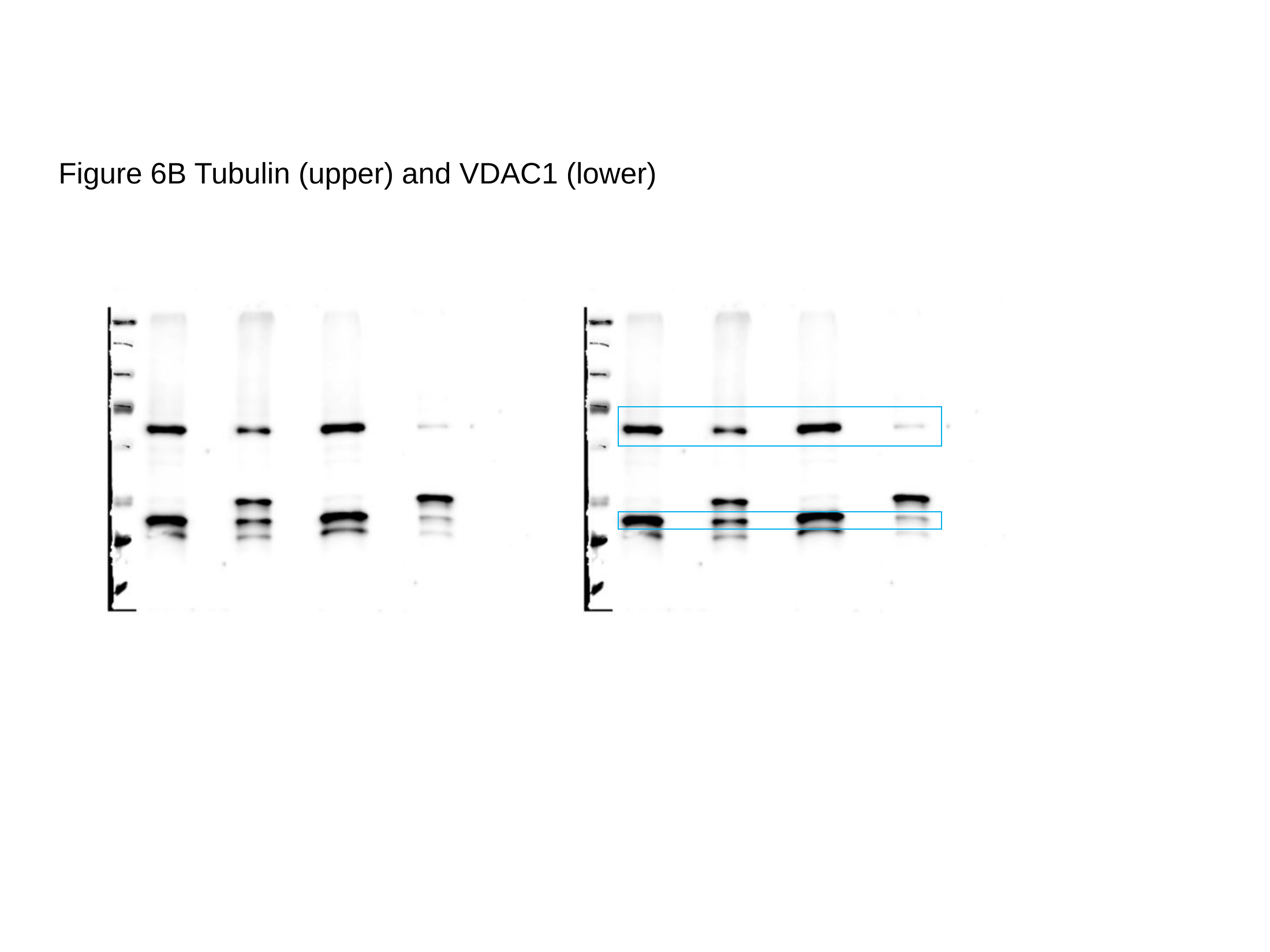

Figure 6B Tubulin (upper) and VDAC1 (lower)

## Slide 9
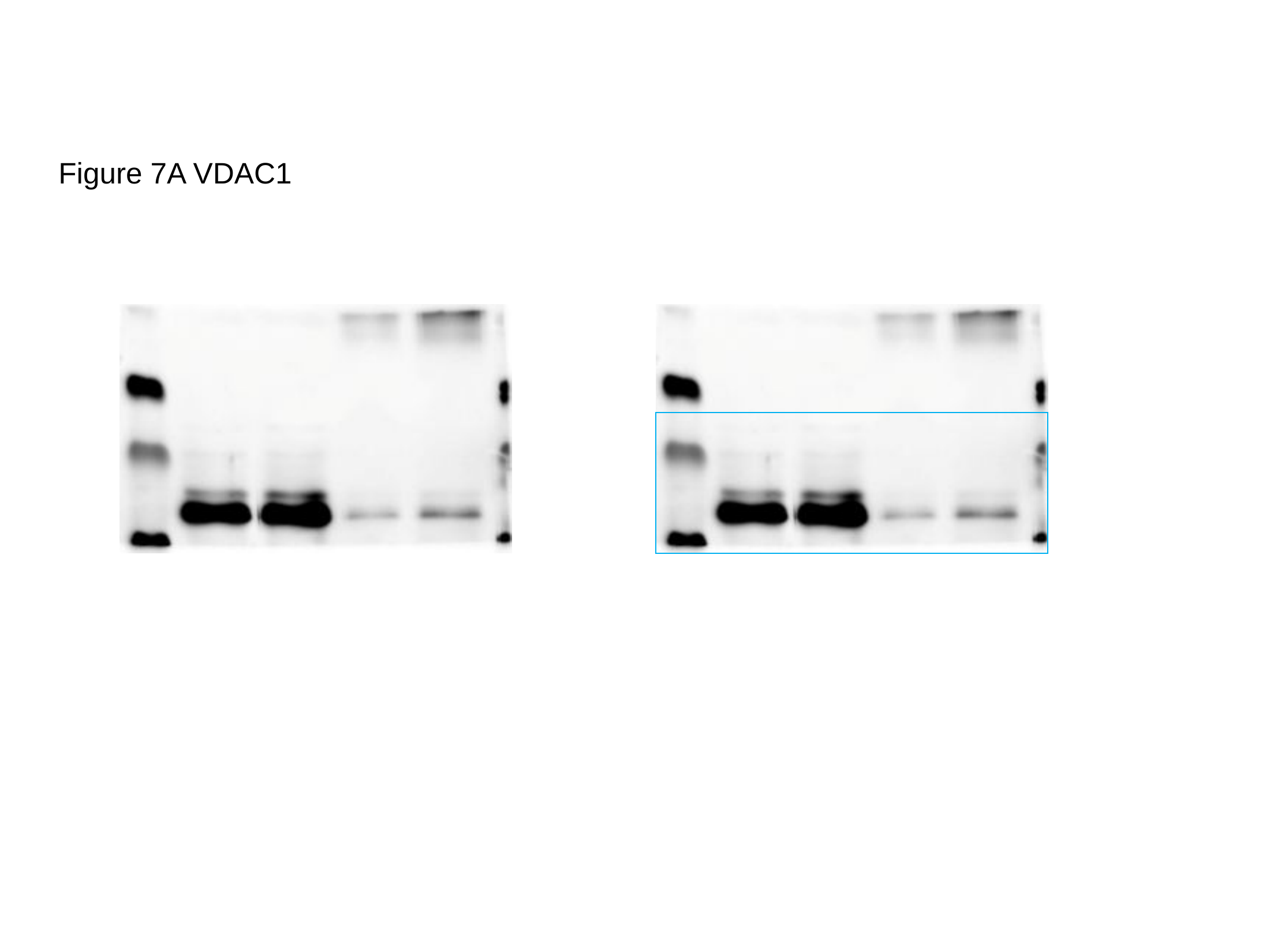

Figure 7A VDAC1

## Slide 10
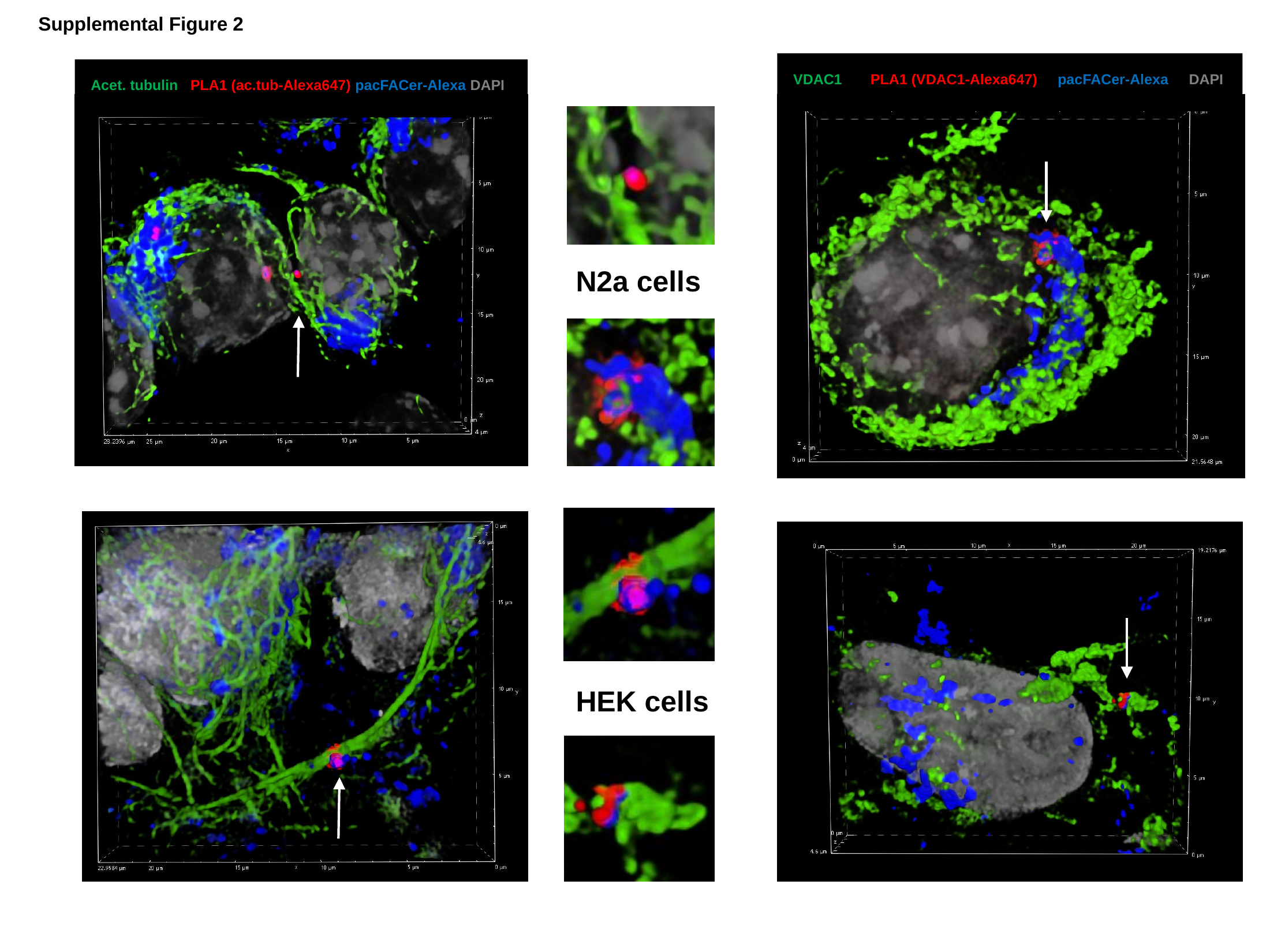

Supplemental Figure 2
 VDAC1 PLA1 (VDAC1-Alexa647) pacFACer-Alexa DAPI
 Acet. tubulin PLA1 (ac.tub-Alexa647) pacFACer-Alexa DAPI
N2a cells
HEK cells

## Slide 11
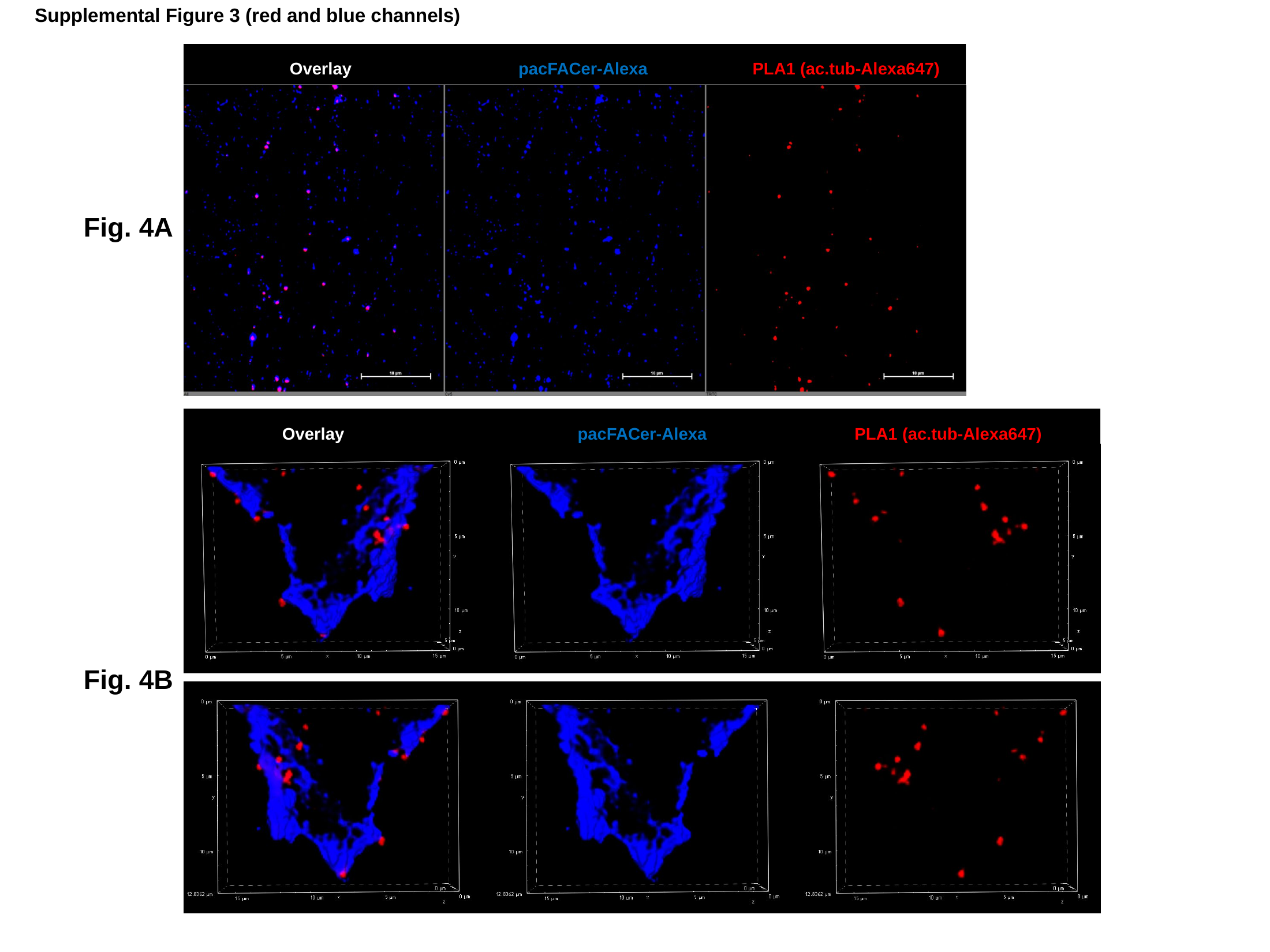

Supplemental Figure 3 (red and blue channels)
 Overlay pacFACer-Alexa PLA1 (ac.tub-Alexa647)
Fig. 4A
 Overlay pacFACer-Alexa PLA1 (ac.tub-Alexa647)
Fig. 4B

## Slide 12
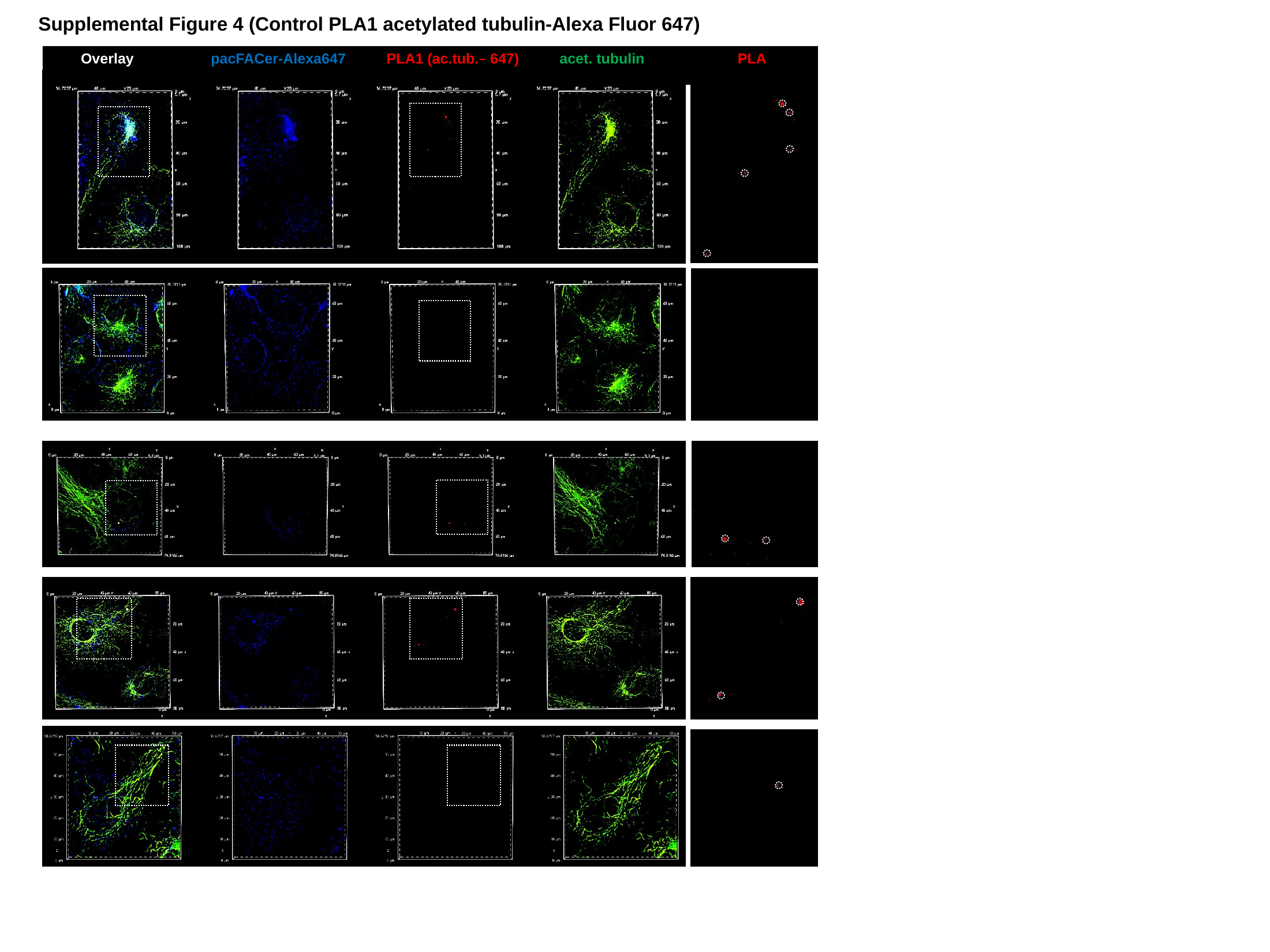

Supplemental Figure 4 (Control PLA1 acetylated tubulin-Alexa Fluor 647)
 Overlay pacFACer-Alexa647 PLA1 (ac.tub.– 647) acet. tubulin PLA

## Slide 13
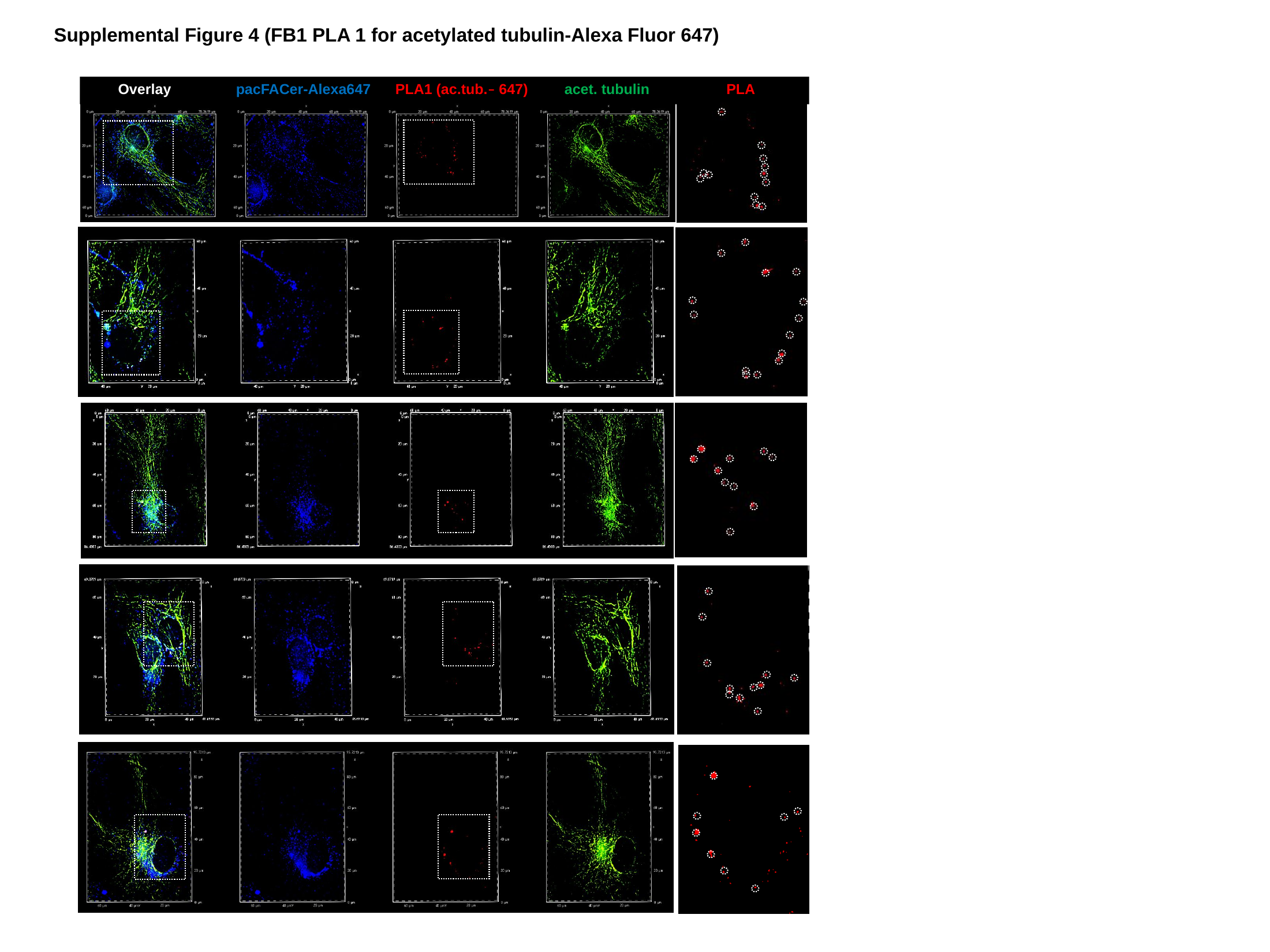

Supplemental Figure 4 (FB1 PLA 1 for acetylated tubulin-Alexa Fluor 647)
 Overlay pacFACer-Alexa647 PLA1 (ac.tub.– 647) acet. tubulin PLA

## Slide 14
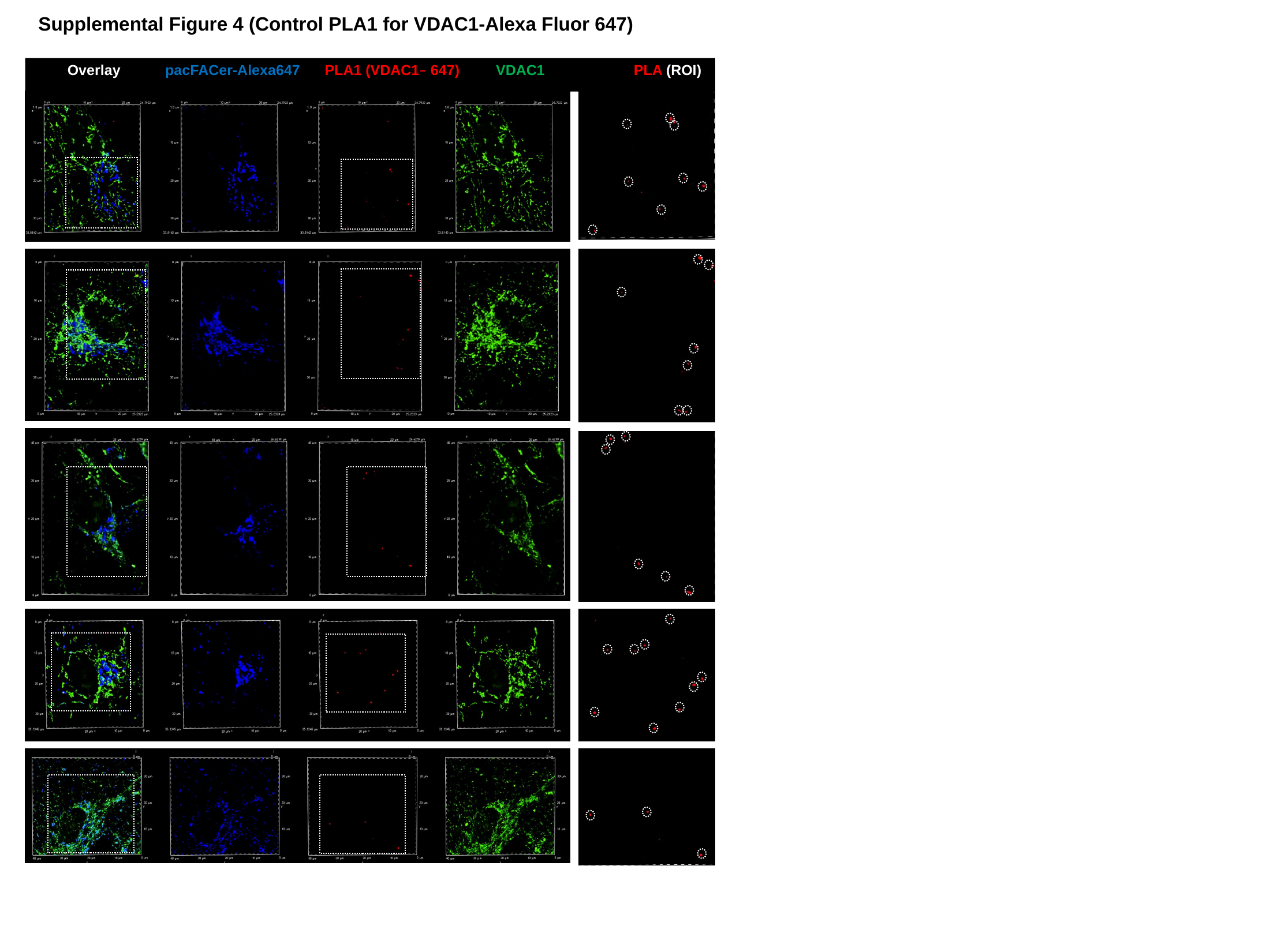

Supplemental Figure 4 (Control PLA1 for VDAC1-Alexa Fluor 647)
 Overlay pacFACer-Alexa647 PLA1 (VDAC1– 647) VDAC1 PLA (ROI)

## Slide 15
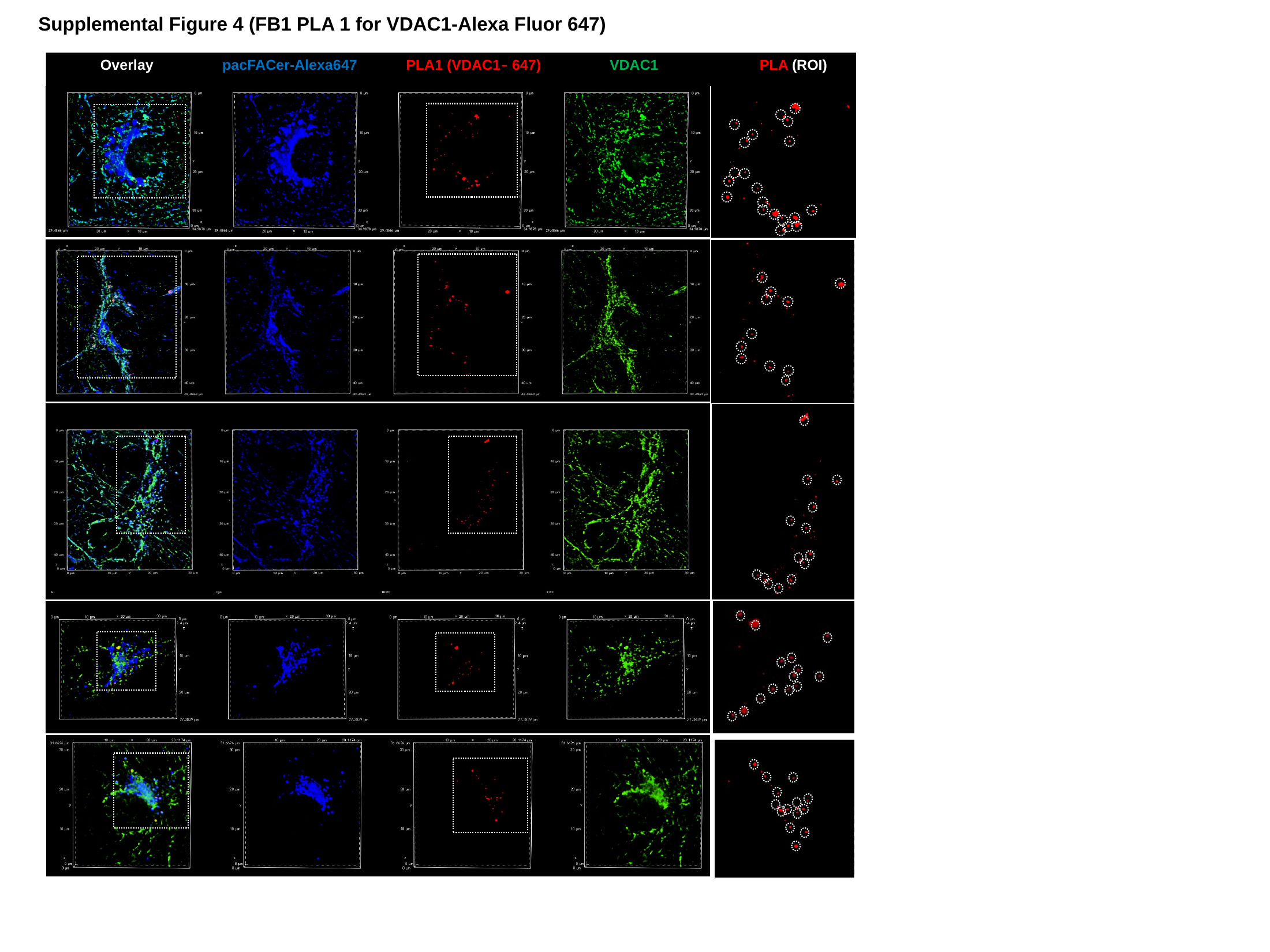

Supplemental Figure 4 (FB1 PLA 1 for VDAC1-Alexa Fluor 647)
 Overlay pacFACer-Alexa647 PLA1 (VDAC1– 647) VDAC1 PLA (ROI)
